# Sustained attention and the flash grab effect

**DOI:** 10.1167/jov.24.2.6

**Published:** 2024-02-21

**Authors:** Nika Adamian, Patrick Cavanagh

**Affiliations:** 1 School of Psychology, University of Aberdeen, Old Aberdeen, UK; 2 Department of Psychology, Glendon College, CVR York University, Toronto, ON, Canada

**Keywords:** motion, motion-induced position shift, attention

## Abstract

When a stationary target is briefly presented on top of a moving background as it reverses direction, the target is displaced perceptually in the direction of the upcoming motion (the flash grab effect). To determine the role of attention in this effect, we investigated whether the predictability of the location of the flash grab target modulates the illusion. First, we established that effect was weaker for spatially predictable targets. Next, we showed that the flash grab effect decreased for a narrower spatial spread of attention before the onset of the target and that it was smaller for left hemifield presentations than right. Finally, we demonstrated that diverting attention away from the target and the background motion decreases the flash grab effect. In the first two experiments, the decrease in the illusion could be attributed to either increased attention to the target or decreased attention to the motion; we assume that increasing attention to the target necessarily decreases attention to the motion. However, in the final experiment, the central task decreases attention to both the target and the motion. The results show a decrease in the illusion and that reveals that attention to the motion is the primary causal factor.

## Introduction

Localization is one of the most important functions of vision, yet it remains poorly understood, especially when a location has to be assigned to a moving object or an object surrounded by motion. A range of motion–position illusions, including the flash drag (Whitney & Cavanagh, 2000), flash lag (Nijhawan, 1994), and flash jump (Cai & Schlag, 2002) vividly illustrate that motion information plays an important role in determining where objects are perceived and shows that motion and position are not processed independently. One particularly powerful example of motion-induced position shifts is the flash grab effect, where an illusory position shift is seen when a target is briefly flashed on top of a moving background that abruptly changes direction (Cavanagh & Anstis, 2013).

**Figure 1.**
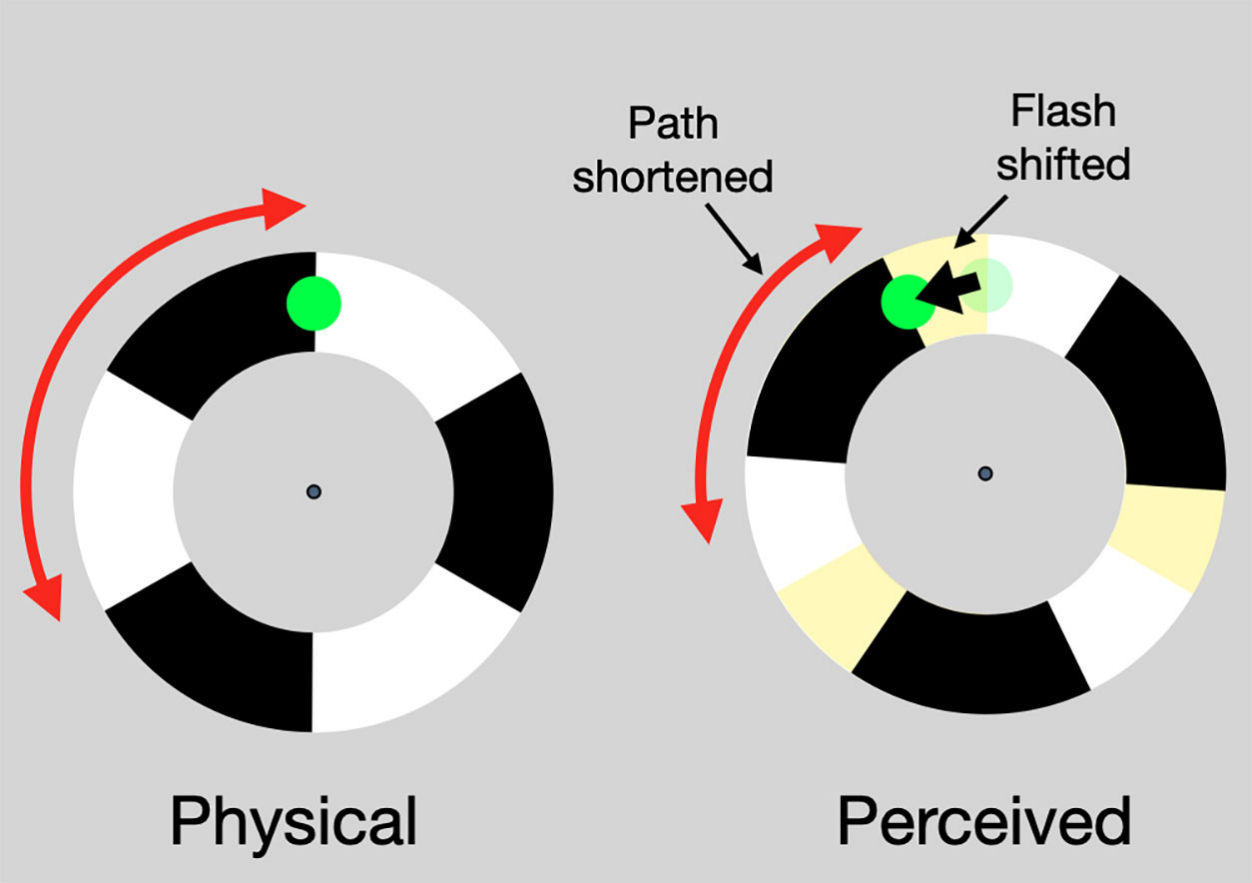
The textured annulus rotates back and forth and when the edge reaches the top, the motion reverses and the green flash appears. The motion path appears shortened, however, and the flash migrates to the edge's perceived location (the flash grab) (Cavanagh & Anstis, 2013). In the experiment here, the rotating texture was *1/f* noise (see Supplementary Movie 1).

The flash grab effect has two components. The first one is the apparent shortening of the background motion trajectory bounded by reversals at both ends, possibly explained by location averaging or predictive position extrapolation (Nijhawan & Khurana, 2010; Johnson et al., 2023; Sinico, Parovel, Casco, & Anstis, 2009). The second component is a position shift of the briefly presented stationary stimulus (flash), which is grabbed to the perceived (shifted) location of the reversal, the end point of the motion trajectory (Figure 1). The illusion is strongest when the transient of the flash coincides with the transient of the motion reversal (Cavanagh & Anstis, 2013), suggesting that the flash grab requires an assumption that the flash belongs to the moving stimulus to inherit its motion and position.

Another important feature of flash grab is the involvement of attention. Cavanagh and Anstis (2013) tested whether the trajectory shortening that underlies the flash grab is still perceived when multiple trajectories are presented. They found that the shortening did not happen for independent, random trajectories when attended as a group; it only happened when a single trajectory within the group could be tracked individually, suggesting that the flash grab requires attention. Tse, Whitney, Anstis, and Cavanagh (2011) used a flash grab stimulus composed of two transparent layers of opposed motion. Participants could switch their attention from one layer of motion to another, and the direction of the flash grab was found to follow the direction of the attended motion. This result proved that attention to motion is sufficient for generating the flash grab effect, even in the absence of any net, low-level motion energy. Both of these studies pointed to the contribution of high-level motion or attentive tracking as an essential part of the illusion.

The studies described manipulated the attentional tracking of the motion. Less is known about the role of attention directed to the target itself in the flash grab. It is well-established that focused spatial attention improves performance compared with distributed attention (Mangun & Hillyard, 1988; Posner, Snyder, & Davidson, 1980) and that attentional benefits decrease with increasing distance from attended location (Downing, 1985; Handy, Kingstone, & Mangun, 1996). Motion-induced position shifts such as the Fröhlich effect (Müsseler & Aschersleben, 1998) and flash lag (Namba & Baldo, 2004; Shioiri, Yamamoto, Oshida, Matsubara, & Yaguchi, 2010; Vreven & Verghese, 2005) have been shown to be reduced by valid attentional cueing. That is, there was less motion-induced shift when attention was already allocated to the space containing the upcoming target. However, in both these illusions, the motion and the target are bound spatiotemporally. For the Fröhlich effect, the motion is that of the target itself, and in the case of flash lag, the location of the flashed target is always judged relative to the location of the moving element. In contrast, for the flash grab (Figure 1), the target location can be manipulated independently from the location of the moving features, allowing us to focus on the effect of attention to the target alone.

**Figure 2.**
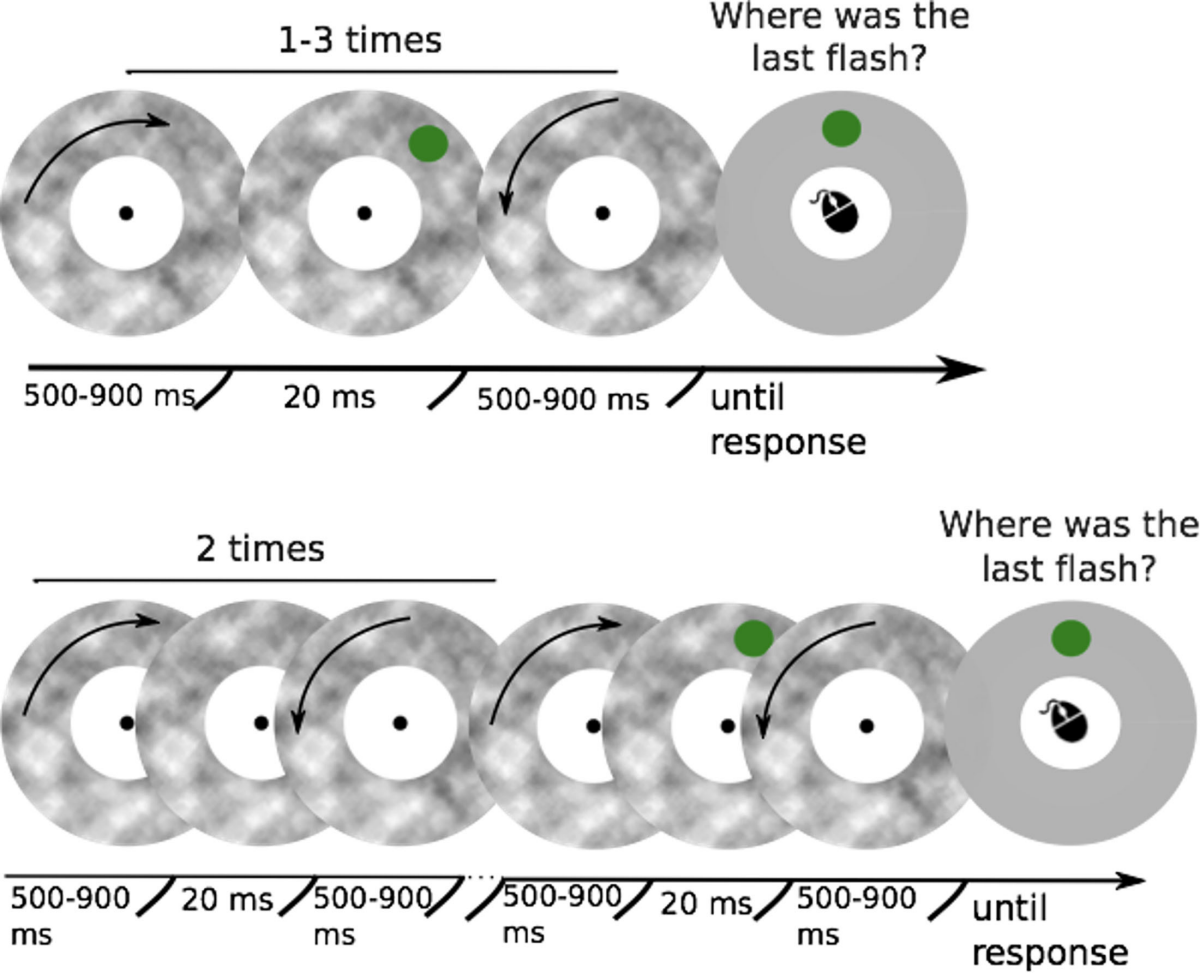
Schematic representation of the procedure used in Experiment 1. The top row corresponds with the trials in first flash, second flash, and third flash conditions. Bottom row corresponds with the trials in time control condition.

Here we examine the attentional modulation of the flash grab effect by varying the spatial predictability or uncertainty of the target location—we assume that the more predictable the target location, the more attention will be focused on its expected location. In all experiments, the task was to observe a rotating texture that rocked back and forth and report the location of a flash that occurred at the moment of a motion reversal by clicking on its remembered location (Figure 2). In Experiment 1, we manipulated spatial predictability by presenting the flash at the same location one or several times. The flash first appeared at an unexpected position that then served as a valid cue to the location of any subsequent flashes. In Experiment 2, participants were cued to a narrow or broad spatial area where the flash would subsequently appear (a single appearance). The cue allowed participants to focus or spread their attention in anticipation of the target. In Experiment 3, we compared the magnitude of the flash grab effect in different regions of the visual field while controlling the spread of attention. Finally, in Experiment 4, we compared the strength of flash grab effect when attention to the motion and the flash was distracted by a second, central task.

We consider three likely alternatives to explain how uncertainty and attention mediate the strength of the flash grab effect. The first is based on the target uncertainty directly and the second on the delay of attention in acquiring the target. The third is based on the effect of attention on the background motion. In the first case, decreased target uncertainty will make its perceived location less susceptible to the effects of the background motion. In other words, the increased predictability will engage attention more strongly to the expected target location, reducing the illusion.

In the second case, it is the delay of attention in reaching the target that produces the position shift and greater target uncertainty will delay the arrival of attention at the target location. This mechanism has been suggested for the Fröhlich effect (Adamian & Cavanagh, 2017; Baldo & Namba, 2002; Kirschfeld & Kammer, 1999; Müsseler & Aschersleben, 1998) and we extend it here to the flash grab stimulus with the assumption again that the flash inherits the motion of the background (Cavanagh & Anstis, 2013). During the time it takes attention to reach the moving target, it continues its inherited motion, producing the mislocalization of its final position when it is acquired. In conditions of low uncertainty, attention is quickly on the target, decreasing this illusory shift.

Finally, in the third alternative, it is the strength of the background motion that controls the illusion and attention to the motion increases the motion strength. The manipulations of target expectation in the experiments affect attention to the target but attention is a limited resource, and this generates a trade-off: increasing attention to the target when its position is highly predictable will decrease attention to the background motion, and vice versa. The physical motion is always the same, but attention to the motion will increase its effectiveness in shifting the flashed test. In the first two experiments here, increased attention to the target is coupled to decreased attention to the motion (and vice versa), preventing us from determining which controls the illusion strength—let alone which of the two possible mechanisms linked to the target is in play (uncertainty or delay). However, the third experiment uncouples the attention to the target and background motion by reducing both with a distracting task. The outcome of the final experiment then favors attention to the background motion as the key determinant of the illusion strength.

## Experiment 1

This experiment measured the flash grab effect when the flash appeared at expected and unexpected locations. To manipulate spatial predictability of the target, we presented the flash at the same location multiple times within the same trial, and asked participants to report the position of the last flash in the trial. We then compared the flash grab of targets presented once (unexpected location) with the flash grab for the last of two or three targets (all at the location of the first). We also included a control condition to measure the contribution of temporal expectations.

### Method

#### Participants

Ten healthy adults took part in the experiment (four males; mean age, 23.2 ± 2.1years; range, 18–29 years). All participants in this and following experiments reported normal or corrected-to-normal vision. All participants gave informed consent in writing before participation and the protocols for the study were approved by the Université Paris Descartes Review Board, CERES, in accordance with French regulations.

#### Stimuli and apparatus

The stimulus was an annulus of 18 degrees of visual angle (dva) outer radius and 14 dva inner radius filled with a five octave, *1/f* noise texture. A new texture was generated for every trial. The annulus was presented against a mid-gray background (10.2 cd/m^2^). A black (2.1 cd/m^2^) centrally located fixation dot remained on screen throughout the experiment. During the trial the annulus rotated back and forth at the speed of 270° (degrees of rotation) per second and changed direction after a variable amount of time (500–900 ms, uniformly distributed between trials). Motion continued for one to three cycles depending on the condition. The starting direction and duration of rotation was chosen randomly for each trial. Once per motion cycle (i.e., on every odd-numbered reversal) the motion stopped for 20 ms, and during that pause a target—a green (19.5 cd/m^2^) disc with a 2 dva diameter—appeared on top of the annulus at 13.5 dva eccentricity (Figure 1).

The experiment took place in an otherwise darkened room. Stimuli were presented on a gamma-corrected, LaCIE Electron monitor (100 Hz; 1,024 × 768 resolution). Participants were seated 57 cm from the monitor with their heads resting on a chin- and headrest. Eye fixation was controlled using EyeTribe eye tracker (The Eye Tribe Aps, Copenhagen, Denmark). The experiment was programmed in MATLAB 8.4 (The MathWorks, Inc., Natick, MA) using the Psychophysics toolbox (Brainard, 1997; Pelli, 1997) for presentation and Eyetribe toolbox (Dalmaijer, 2014) for eye tracking.

#### Procedure and design

Participants performed 240 trials in 6 blocks, 60 trials per condition (as described elsewhere in this article). Before the experiment participants performed 20 trials identical to the experimental trials as a practice. The eye tracker was calibrated using a standard nine-point calibration procedure before starting the experiment. Gaze position was collected throughout the trial at 60 Hz (binocularly), and trial was interrupted and presented later if the gaze was detected outside of the fixation window (0.5 dva around fixation).

The procedure for the experiment is shown in Figure 2. The beginning of each trial was triggered by participants fixating their gaze on the central point. Then, the textured annulus appeared on the screen and immediately started moving back and forth.

There were four conditions. In the first flash condition, the target was presented at a random location during the first reversal of the texture. Thus, neither the location of the target nor the exact timing of the reversal was known by the participant beforehand. In the second flash and third flash conditions, the target was presented two or three times, respectively, without a change in location, during consecutive motion cycles. In these conditions, both the time of the reversal and the location of the target became expected after the first motion cycle. In the time control condition there were three back-and-forth rotations of the texture, but the target was only presented in the last one, at a random location. Thus, the timing of the reversal but not the location of the flash could be anticipated. Trials in these four conditions were presented in random order.

Participants reported the perceived location of the last target of the trial using a mouse cursor that could be moved around the screen at the same eccentricity as the target. Participants were aware that the targets within the trial were typically presented at the same location (except for the control condition).

### Results

The magnitude of the flash grab effect was reported as the smaller of the clockwise vs. counterclockwise differences between the physical position of the target and the position reported by the subject, in degrees of arc (e.g., −90° instead of 270°). Trials with deviant responses were removed using the median absolute deviation approach (Leys, Ley, Klein, Bernard, & Licata, 2013), resulting in the removal of 4% of trials. Results from trials with counterclockwise (negative) direction of the expected position shift were flipped in sign. The reported direction of the effect matched the expected direction in 96% of the trials, suggesting that the stimulus successfully produced the flash grab effect.

**Figure 3.**
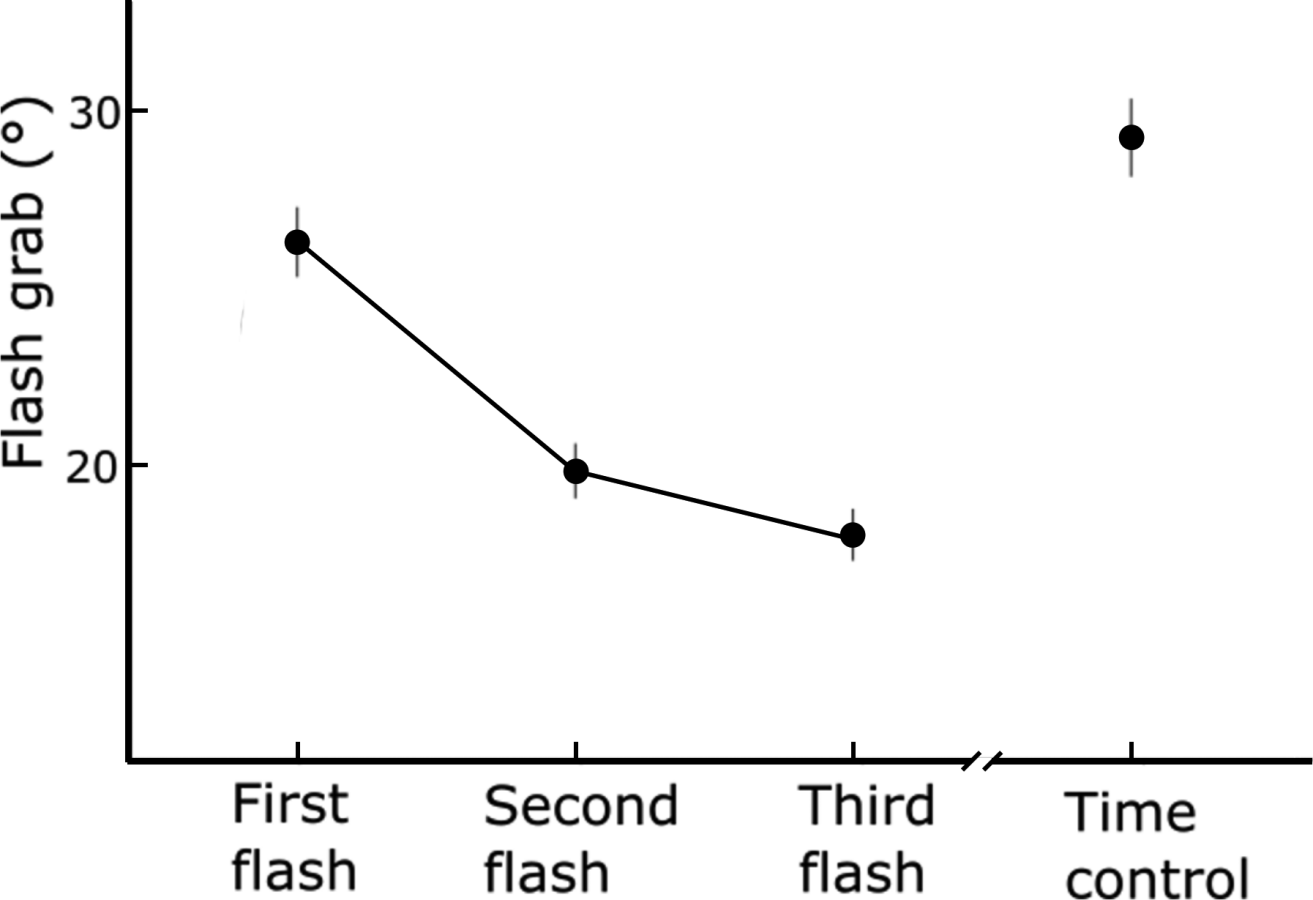
Averages of flash grab effect for the four conditions in Experiment 1. Error bars represent within-participants 95% confidence intervals.

Condition means were submitted to a repeated-measures analysis of variance. Beyond the overall effect of target location certainty, we were interested in the following pairwise comparisons: first flash vs. second flash, as well as second flash vs. third flash to capture the effect of increasing spatial certainty, and first flash vs. time control for temporal certainty. Figure 3 shows the mean flash grab shifts for each condition.

**Figure 4.**
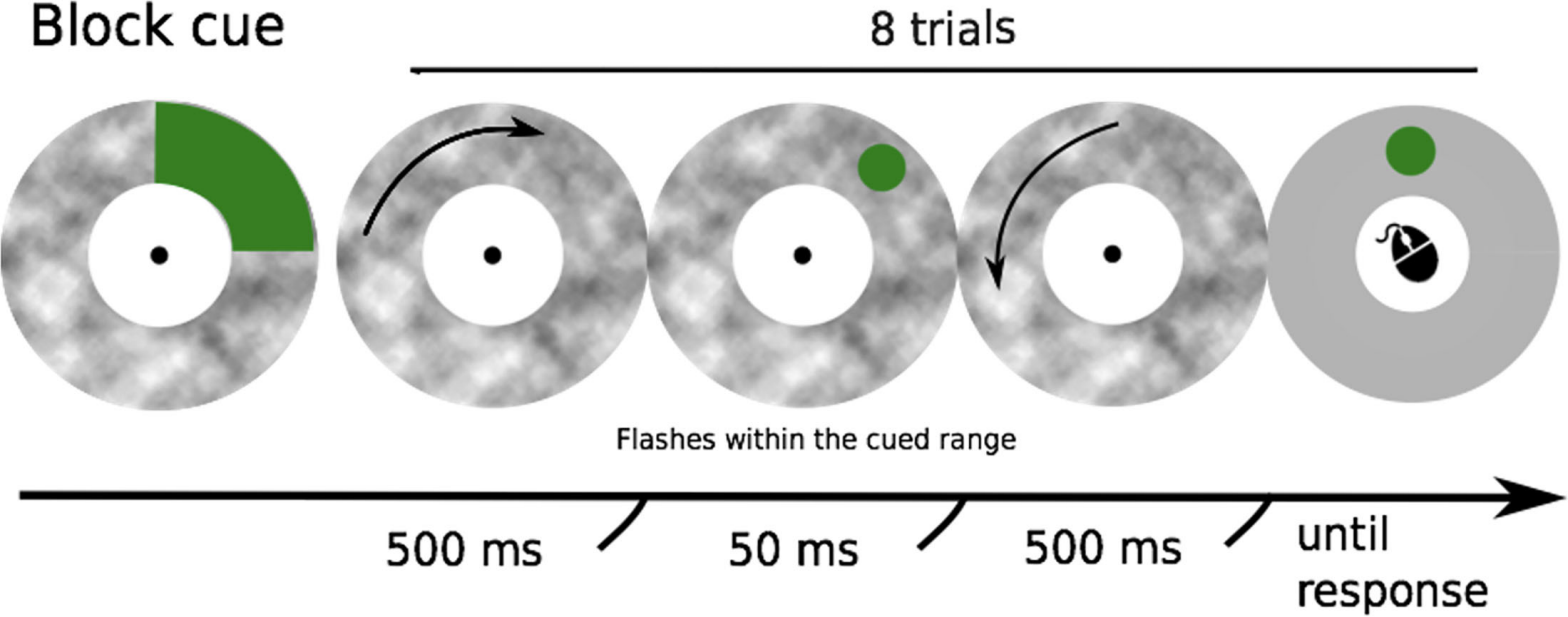
Schematic representation of the procedure used in Experiment 2. Each block of eight trials began with the presentation of a spatial cue. The green area of the cue indicated the range of possible target locations in the following block. Cues could cover 360°, 180° (left or right hemifield, 90° (upper left, lower left, upper right, lower right quadrants), 45° (in the middle of each quadrant), or 10° (precisely indicating the location of the target). Each trial consisted of 1,000 ms of motion reversing direction after 500 ms. At the reversal, the motion stopped for 50 ms during which the target was presented.

The results show that the flash grab magnitude was significantly affected by the condition, *F_(3, 27)_* = 156.53, *p* < 10^−15^, *η^2^* = 0.47 (degrees of freedom corrected according to the Greenhouse–Geisser procedure). Post hoc pairwise comparisons with Holm's correction showed that the effect decreased after the first presentation of the target, first flash vs. second flash, *t_(9)_* = 11.40, *p* < 10^−5^, as well as after the second presentation of the target, second flash vs. third flash, *t_(9_*_)_ = 2.33, *p* = .045. The effect increased to its largest magnitude for the control condition, first flash vs. time control, *t_(9)_* = −4.44, p = 0.002. In both the first flash and the time control conditions, the participants reported the position of the first and only flash, but in the control condition, the timing was well-established after several regular reversals of the background motion. In both these conditions, unlike the second and third flash conditions, the position was unknown.

### Discussion

Experiment 1 explored how the flash grab effect changed depending on the available spatiotemporal information about the upcoming target. We manipulated spatial predictability by presenting the target once or multiple times at the same location, and we manipulated temporal predictability by presenting the target either at the first, unexpected reversal, or after three identical back-and-forth oscillations of the background.

The main finding is that a flash presented at an unexpected location is shifted or grabbed more than a flash whose location is known in advance. In other words, narrowing the focus of attention to the expected location reduced the influence of motion on the perceived position of the flash. Numerous articles on spatial attention suggests that adjusting the focus of attention to a smaller area improves perceptual performance at the attended location (Castiello & Umiltà, 1990; Eriksen & James, 1986; Eriksen & Murphy, 1987; Mangun & Hillyard, 1988). In our case, increased attention resulted in more veridical perception of flashed target location. This is in line with a number of studies showing a decreased flash lag (Namba & Baldo, 2004; Shioiri et al., 2010; Vreven & Verghese, 2005) or Fröhlich effect (Müsseler & Aschersleben, 1998) when valid spatial cues were used. The effect size for the flash grab shift here (approaching 30° in the control condition) is significantly larger than in previous experiments (e.g., ∼10°) (Cavanagh & Anstis, 2013), where the test flash was presented continually at the same location throughout the experiment. It could be argued that each repetition provides additional sensory evidence about the target's location, bringing the response closer to the veridical value, reducing the illusion. However, there is no reason to assume that the additional samples have any access to the veridical value; they should all be as distorted as the first and so result only in a reduced variability. The successive samples can only become more veridical if the sampling process is changed. And that is what the increased attention does as it narrows the range over which attention is allocated.

Although it is possible that the increased attention to the target location reduced the illusion, one consequence of increasing attention to the target is a decrease of attention to the background motion which also may reduce the illusion. Because of this trade-off, we cannot distinguish between the effects of attention to the target versus to the background motion; we can only state that manipulating attention has a strong effect on the illusion.

The position of the target was always constant within the trial and thus fully disclosed after one appearance; nevertheless, the flash grab effect was further reduced at its third presentation compared with the second presentations. This pattern of results suggests a continued tightening of the attentional distribution between the consecutive presentations of the flash. One possibility is that narrowing of the attentional focus continues as more information about the position of the target is accumulated with each consecutive presentation. Additionally, shifting the focus of attention toward the second, more veridically perceived location of the target could also improve the spatial allocation of attention. It is known that attentional benefits decrease as a function of the distance from the focus of attention (Downing, 1985; Eriksen & James, 1986; LaBerge, 1983). Therefore, the closer the focus of attention is to the physical target location, the more the target will be protected from the motion-induced shift on the next presentation. This dynamic is explored in greater detail in Experiment 2.

Finally, we unexpectedly observed a significant increase of the flash grab illusion when a single flash was presented after several back-and-forth oscillations of the background. One possibility is that the time control condition created a temporal expectation that the flash would occur during the third back-and-forth rotation and this temporal expectation increased the flash-grab effect (Coffey et al., 2019). Another factor that could have increased the motion-induced position shift is that in the absence of the target, motion attracted more attention, decreasing attention to the target feature when it appeared at an unknown location. This possibility is further explored in Experiment 4. Additionally, there may be a response bias of the last seen motion that affects the mouse click on the remembered flash location, pulling it in the direction of that motion. Although we cannot rule out this bias, its effect will be constant, with changes in the number of repetitions before the response and so should cancel out when looking at differences across conditions.

## Experiment 2

Experiment 1 demonstrated that targets at expected locations are less subject to the flash grab effect. Here we extended this finding by asking whether the strength of the effect is modulated by the spatial uncertainty of the target. In this experiment, trials were presented in blocks of eight. Before each block, a cue indicated a range of possible target locations, prompting participants to focus or distribute their attention to better localize the target. Based on the results of Experiment 1, we expected that the flash grab effect would decrease in blocks with more precise attention cues.

### Method

#### Participants

Fifteen healthy adults (6 males; mean age, 20.9 ± 2.2 years; range, 18–26 years) were recruited for Experiment 2. All gave written informed consent before the start of the experiment and received monetary compensation for their time. Five participants were experienced psychophysical observers; two of them had participated in Experiment 1.

#### Stimuli and apparatus

The equipment was identical to that of Experiment 1. The flash grab stimulus was similar to the one used in Experiment 1 with the following changes. In all the trials, the motion duration was fixed: the motion reversed direction after 500 ms (having travelled 135° of rotation). There was one reversal per trial, always coinciding with the target presentation. The starting direction of motion was randomly chosen on a trial-by-trial basis.

#### Procedure and design

Trials were presented in blocks of eight. A cue at the beginning of each block indicated the area where the targets would appear (Figure 4). The exact physical position of the target was selected randomly from a uniform distribution within the cued range on a trial-by-trial basis. There were five conditions based on the range of target locations: 1) 360° (targets appeared anywhere around the circular background); 2) 180° (all targets appeared either in the left or in the right hemifield), 3) 90° (all targets appeared within one of the quadrants of the visual field (upper left, lower left, upper right, or lower right); 4) 45° (all targets appeared along a 45° arc centered within one of the quadrants); and 5) 0° (all targets appeared at a fixed location). Targets were presented with a fixed eccentricity of 13.5 dva.

Participants were asked to attend the cued area while maintaining central fixation and report the location of the target on each trial. They were instructed to report the perceived location of the target, even if it appeared to fall outside the cued range (these shifts outside the cued range were caused by the illusion, there were never any locations presented physically outside the cued range). There were 8 blocks per condition totaling 320 trials. Across all blocks, each hemifield and quadrant was represented with equal probability.

As in Experiment 1, responses were converted into flash grab estimates by subtracting the physical positions of the target from the reported positions in each trial and reversing the sign in those trials where negative (counterclockwise) shift was expected. Outliers (3.5% of all the trials) were removed using the median absolute deviation approach. Of the remaining 4,634 trials 96.8% showed the illusion in the expected direction.

### Results

**Figure 5.**
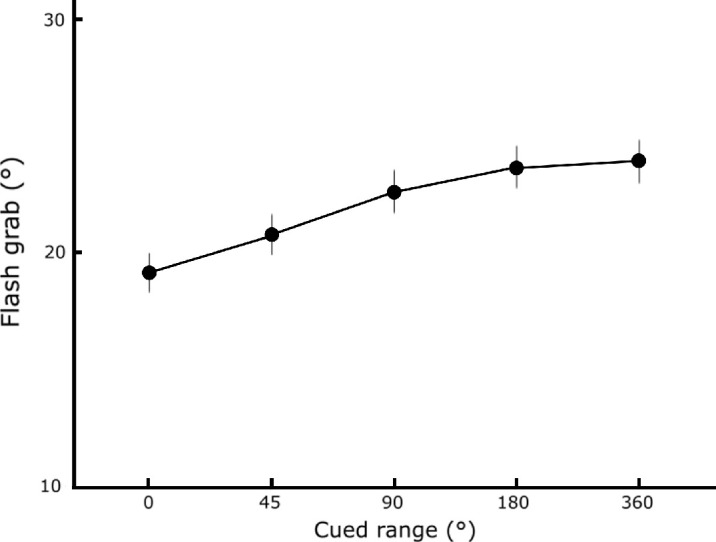
Mean flash grab magnitude in each cueing condition. Error bars are within-participant 95% confidence intervals.

The increase in spatial range of targets resulted in the increase of the illusion, as shown in Figure 5, *F_(4, 56)_* = 11.78, *p* < 10^−6^, *η^2^* = 0.06. This increase in the flash grab effect with increasing spatial uncertainty is consistent with the results of Experiment 1. Consecutive comparisons between cueing conditions did not reach statistical significance, 0°–45°: t_(14)_ = 2.03, *p* = 0.06; 45°–90°: t_(14)_ = 2.17, *p* = 0.05; 90°–180°: t_(14)_ = 0.86, *p* = 0.41; 180°–360°: t_(14)_ = 0.97, *p* = 0.35.

### Discussion

In Experiment 2, we manipulated the spatial range of attention to quantify the reduction of the flash grab illusion for expected targets. A similar paradigm has been used previously to demonstrate that decreasing certainty regarding the location of the target results in slower reaction times and decreased accuracy in a detection task (Mangun & Hillyard, 1988; Voytek et al., 2017), consistent with the effects of endogenous spatial attention. Our results confirmed that a narrower attentional distribution results in a decreased flash grab effect (greater accuracy), as was found in Experiment 1. Once again, though, these results do not distinguish between the effect of increased attention to the target versus decreased attention to the background motion. Either or both may have caused the decrease in the illusion strength.

## Experiment 3

If the flash grab illusion depends on the distribution and availability of attention, it could also be susceptible to attentional asymmetries. There are many studies suggesting a left–right asymmetry in attention (Holländer, Corballis, & Hamm, 2005; Matthews & Welch, 2015; Verleger et al., 2009), but this effect is often attributable to lateralization of function specific to individual tasks that either require more verbal or more spatial processing (Asanowicz, Marzecová, Jaśkowski, & Wolski, 2012), or to reading direction (Ransley, Goodbourn, Nguyen, Moustafa, & Holcombe, 2018). Nevertheless, there is compelling evidence of a mild leftward bias in spatial attention referred to as pseudoneglect (Bowers & Heilman, 1980; Gigliotta, Malkinson, Miglino, & Bartolomeo, 2017). For example, in a line bisection task, healthy individuals will bisect a line to the left of center (Jewell & McCourt, 2000), and this effect is attributed to the overestimation of the left half owing to a left side attentional bias. An advantage of lower visual field over the upper field (Levine & McAnany, 2005) has also been reported, especially for motion and spatial judgments (Amenedo, Pazo-Alvarez, & Cadaveira, 2007; Lakha & Humphreys, 2005). In contrast with these findings of spatial asymmetries, Carrasco, Talgar, and Cameron (2001) claimed that, when visual factors are controlled, attentional effects do not vary as a function of location. Here we measure the flash grab within a 90° spatial range located in the different parts of the visual field to link the illusion to potential attentional mechanisms.

### Method

#### Participants

Twelve right-handed healthy adults (5 males; mean age, 22.4 ± 2.4; range, 16–30 years) were recruited for Experiment 3. All gave written informed consent before the start of the experiment and received monetary compensation for their time. None of them participated in the previous experiments.

#### Stimuli and apparatus

The stimuli and experimental set up were identical to those of Experiment 2.

#### Procedure and design

The trial structure was identical to the Experiment 2. In all the blocks the target range was 90°. This 90° area could be positioned at one of the eight overlapping locations around the moving texture starting at 0° (upper right quadrant) to 315° (top quadrant) in steps of 45°. Thus, we measured flash grab in target ranges located within the quadrants of the visual field (starting at 0°, 90°, 180°, and 270°) or centered on the meridians (starting at 135° and 315°, the vertical meridian; or 45° and 225°, the horizontal meridian). There were 40 trials per location, delivered in blocks of 8 trials. Data were treated in the same way as in Experiment 2.

### Results

**Figure 6.**
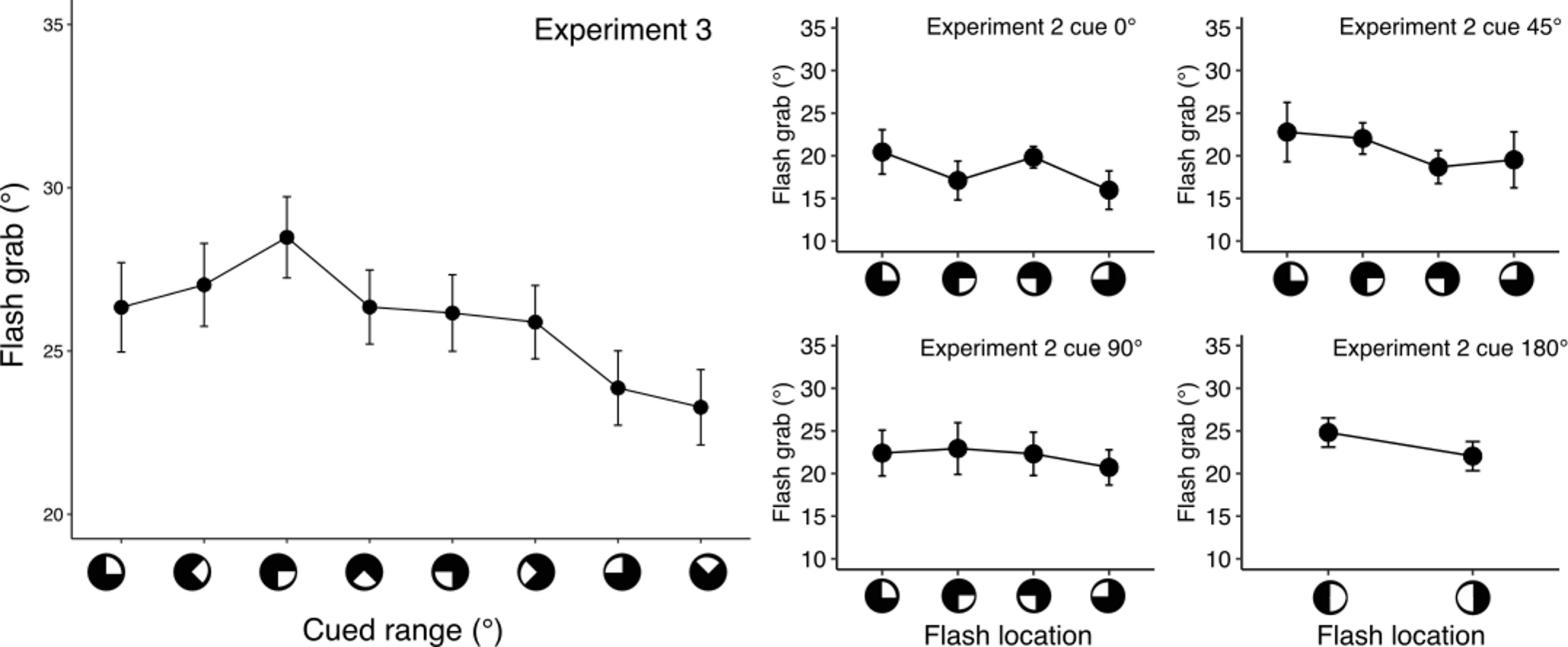
Average flash grab magnitude in each of the cued ranges in Experiment 3. In the right hand panels, a similar analysis is also shown for the data from different cued locations in Experiment 2. Error bars are within-subject 95% confidence intervals.

Figure 6 shows the flash grab illusion reported in each of the cued 90° ranges. After regrouping of the data across various 90° ranges (Table 1), we also compared flash grab in the attended areas within and across quadrants (crossing a meridian or not), in the upper (starting at 0°, 270°, and 315°) and lower (starting at 90°, 135°, and 180°) visual fields, as well as in the left (180°, 225°, and 270°) and right (0°, 45°, and 90°) visual fields.

**Table 1.** Results of Experiment 3.

Location	Flash Grab (°)	SE	CI
Within quadrants	25.73	0.4	[24.92–26.5]
Across quadrants	26.24	0.42	[25.74–27.4]
Left visual field	25.33	0.42	[23.76–25.42]
Right visual field	27.31	0.4	[26.56–28.14]
Upper visual field	24.55	0.39	[23.73–26.57]
Lower visual field	26.98	0.44	[26.45–28.17]

CI, confidence interval; SE, standard error.

We did not find a statistically significant difference in flash grab in quadrants located between versus across vertical and horizontal meridians, t*_(11)_* = −1.24, *p* = 0.24, or in upper versus lower visual hemifields, t*_(11)_* = 1.53, *p* = 0.15; *p* values corrected for multiple comparisons using Holm method. However, the illusion was weaker in the left hemifield compared with the right hemifield, t*_(11)_* = 2.6, *p* = 0.026. Given the modest size of this lateral asymmetry, we attempted to confirm the finding using the dataset from the Experiment 2; more specifically, trials from conditions where cued areas were restricted to a hemifield, a quadrant, or a specific location (Figure 6, right). In these conditions, participants were presented with at least 10 trials in each of the four quadrants (or 2 hemifields in case of 180° cue). The flash grab effect was weaker for cueing in the left hemifield than in the right when the cueing range spanned 180°, *t*_(14)_ = −2.46, *p* = 0.03; 45°, *t*_(14)_ = −2.174, *p* = 0.047; or when the cue indicated the exact location of the upcoming flash, *t*_(11)_ = −2.96, *p* = 0.01, but not when the cueing range spanned 90°, *t*_(14)_ = −0.8, *p* = 0.43. The difference between upper and lower hemifields did not reach statistical significance in any of the re-analyzed conditions: 90°: *t*_(14)_ = 0.98, *p* = 0.35; 45°: *t*_(14)_ = −0.64, *p* = 0.53; 0°: *t*_(11)_ = 0.62, *p* = 0.55.

### Discussion

In Experiment 3, we measured the flash grab effect within attended regions of constant size (90°) situated in different parts of the visual field to determine if there were variations in the magnitude of the illusion as a function of location and whether this could be attributed to spatial variations in the effectiveness of attention. We hypothesized that the illusion would be weaker in locations associated with more effective attentional processing. Indeed, the data showed that flash grab was weaker in the left hemifield (responses were more veridical) compared with the right hemifield, both in Experiment 3 and in a post hoc analysis of Experiment 2. This result is consistent with the findings of a mild left bias in spatial attention, pseudoneglect, in healthy individuals (Bowers & Heilman, 1980). The evidence for an upper versus lower field asymmetry did not reach significance.

Interestingly, patients with right parietal damage and left side neglect also show a weaker illusion on the left side (De Vito et al., 2015). However, in these patients, attention is impaired on the left side, reversing our claim that more effective attention should produce a weaker illusion. Importantly, in neglect patients, attention will be reduced to both the flashed target and the background motion on the left side. This finding suggests that the decrease of attention to the target may not be the critical factor for the flash grab effect. Instead, it may be the decrease of attention to the motion that controls the strength of the illusion. We could not separate the two effects in Experiments 1 and 2, because, in those experiments, increased attention to the target would be accompanied by decreased attention to the motion owing to the attentional trade-off of limited attentional resources. To disentangle the contributions of attention to the target and attention to the motion, we next simulate the loss of attention to both that occurs in neglect patients but using a central distracting task in healthy participants.

## Experiment 4

The results of Experiment 1 showed a strong flash grab effect when participants observed the moving texture without any targets presented for the first few reversals, leading to the possibility that increased attention to the moving background may increase its effect on the shift of the target's perceived location. To test this hypothesis, we adapted Mack and Rock's (1998) unexpected test paradigm to the flash grab stimulus. We used an online, between-group experiment in which one group of participants was presented with the flash grab stimulus while performing an unrelated task at fixation. The central task directed their attention away from the moving part of the stimulus, and the flash was completely unexpected. The other group of participants also did not expect the flash, but their only task was to observe the moving texture. The central task reduces attention to both the flashed target and the motion, breaking their inverse coupling that held in the previous experiments. If attending to the motion determines the illusory displacement then the flash grab effect will decrease for the group with the central task and less attention to the motion, whereas if attending to the target determines the displacement, then the flash grab effect will increase for the group with the central task and less attention to the target.

This experiment was preceded by a pilot study in which we presented participants with the flash grab stimulus in the online setting and established that participants experienced flash grab effect (31/34 participants reported perceiving the shift in the direction of motion after the flash).

### Method

#### Participants

Participants were recruited through Prolific (www.prolific.co). Two hundred fifty adults were recruited for the version of the experiment with the central task, and a further 100 participants were recruited for the version without the central task. Data from three participants from the first group were lost owing to a technical issue. All participants gave informed consent before the start of the experiment and received monetary compensation for their time. The study was approved by the Ethics Committee of the School of Psychology in the University of Aberdeen.

#### Stimuli and apparatus

**Figure 7.**
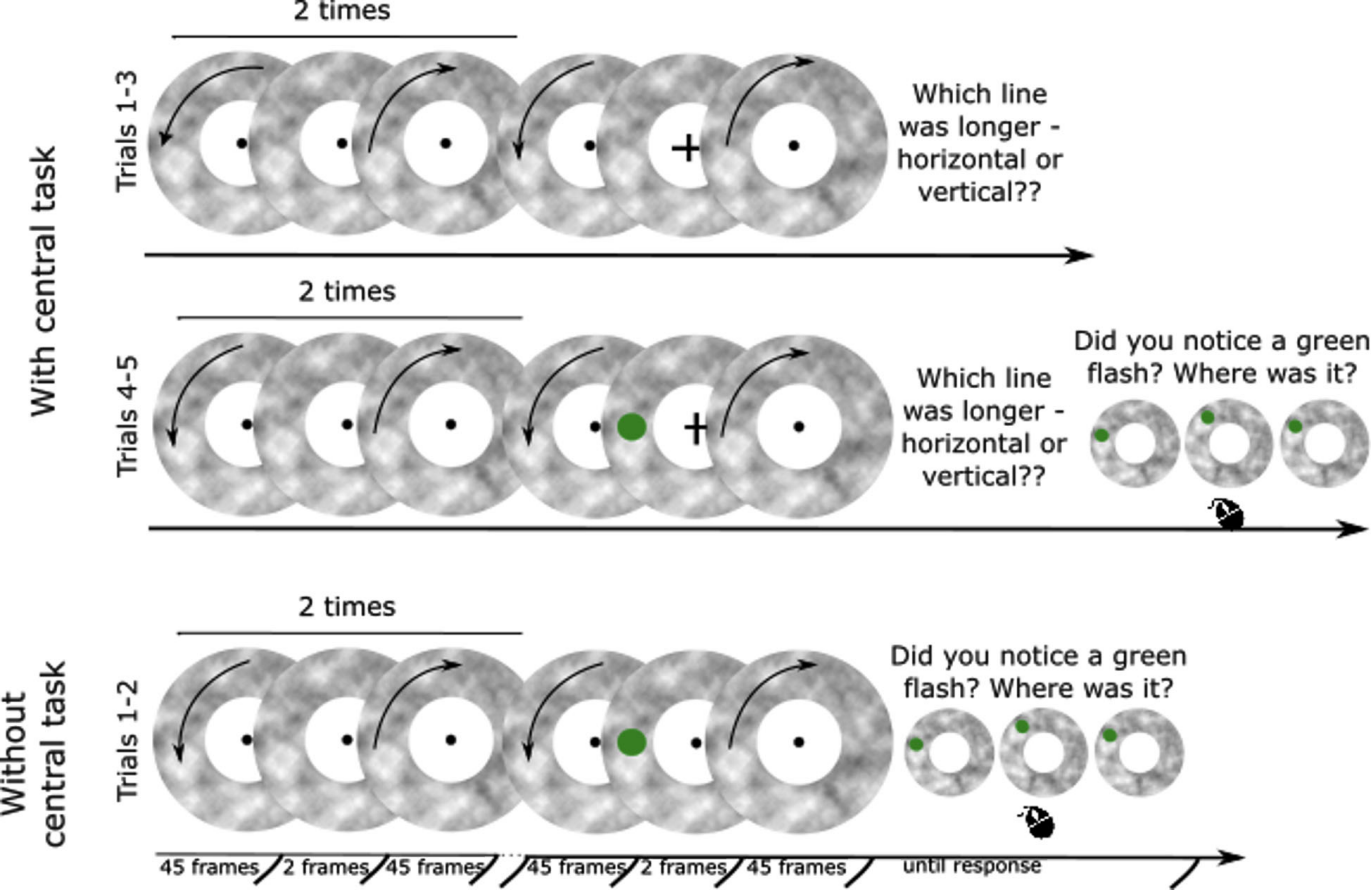
Schematic representation of the procedure used in Experiment 4.

Participants could only participate in the study from a desktop computer with monitor running at a 60-Hz frame rate. The online experiment was created in PsychoPy (Peirce et al., 2019) and was hosted on Pavlovia. Because screen sizes differed between participants, stimuli sizes were expressed in PsychoPy's height units and stimuli were scaled to participants’ screens. The stimulus was very similar to the one used in Experiments 1 and 2 (see Figure 7), but the procedure was adapted to resemble the inattentional blindness paradigm (Mack & Rock, 1998). Each participant was presented with the target twice (the first time being fully unexpected), and in all cases the target was presented at the same location, on the left-hand side of the moving texture. In addition, in the version of the experiment with the central task, a fixation dot turned into a fixation cross during one of the motion reversals. The fixation cross was presented for two frames (33 ms) and one of its components (horizontal or vertical) was 20% longer than the other.

**Figure 8.**
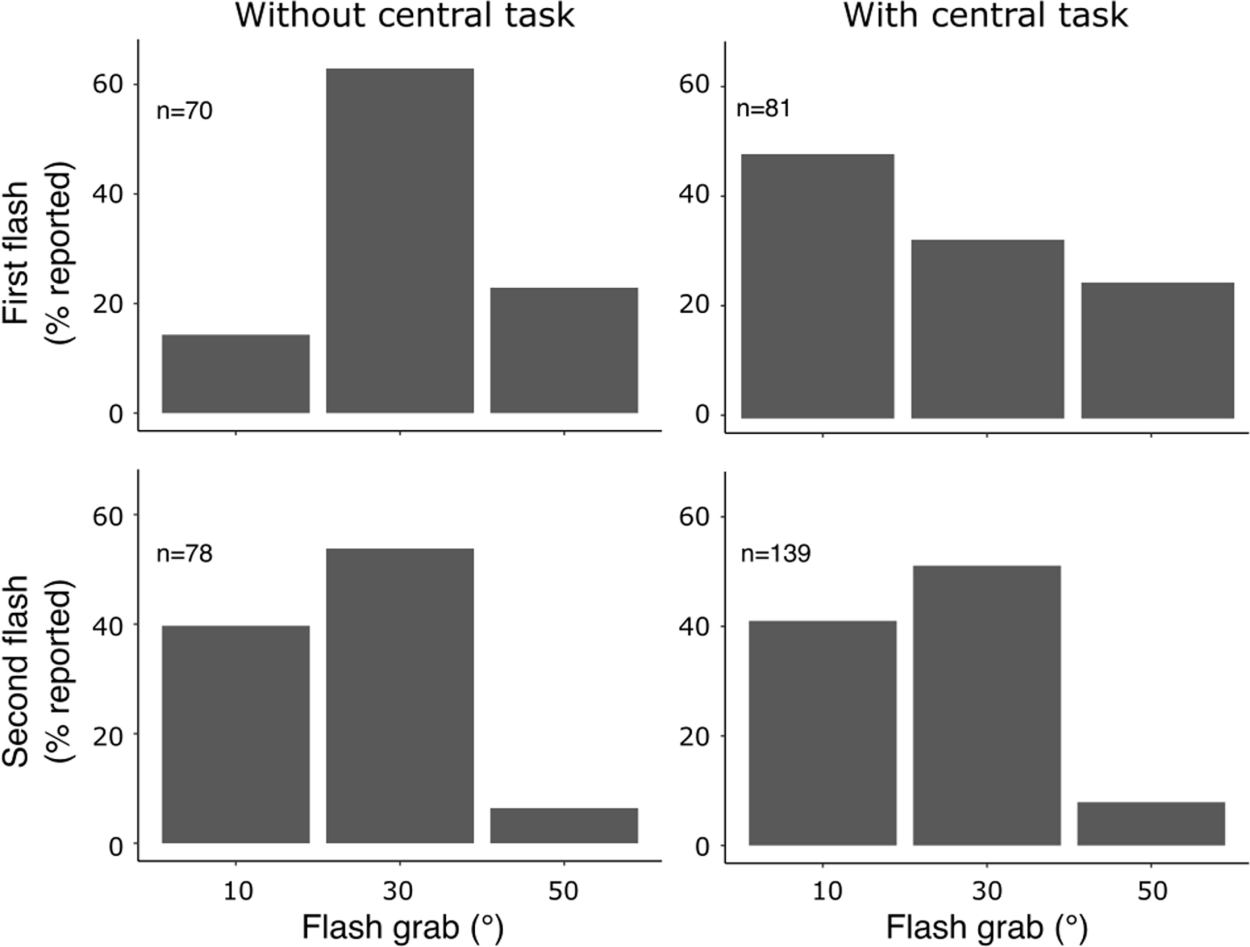
Distribution of flash location responses among participants who reported noticing the flash during the first (top row) and second (bottom row) presentation of the flash in the condition with the central task (left column) and without (right column).

#### Procedure and design

The group of participants who took part in the version of the experiment with the central task completed five trials in total. They were instructed to fixate on the dot in the center of the screen and judge whether the horizontal or the vertical component of the briefly centrally presented cross was longer. They were not aware that they would be presented with a target or that they would need to report its location. In each trial, they were presented with the textured annulus that oscillated back-and-forth three times starting in a counterclockwise direction. On the third back-and-forth rotation, the central task appeared during the reversal. After each trial, participants responded which side of the cross was longer with a mouse click. On trial 4, the target (a green disk) unexpectedly appeared on top of the moving texture at the same time as the central task. After this trial, participants were asked whether they noticed the target and where was it located. The location question had three response options, and participants had to choose which image most closely resembled what they perceived. The response options had the green disk shifted 10°, 30°, or 50° clockwise, with the order of the options randomized. The final trial was the exact repetition of trial 4, but participants were now aware of the possibility of the green disk appearing.

The version of the experiment without the central task had two trials only, both with the flash present. Participants were instructed to fixate in the centre of the screen and observe the display. They were told they will be asked to describe what they saw. The trials were identical to trials 4 and 5 of the first group but did not include the central task.

### Results

Of the 247 participants in the group with the central task, 206 (83.4%) gave the correct response to the central task on the trial when the flash was presented and were considered sufficiently distracted from the moving texture. After the first presentation of the flash, 81 (39.3%) reported noticing the target. From this subgroup, 37 (45.7%) reported that the closest position to what they perceived was the flash shifted 10° from its veridical position, 25 (30.9%) reported perceiving the flash shifted 30°, and 19 (23.5%) reported perceiving the flash shifted 50° (Figure 8). Overall, the smallest shift was chosen more often, χ^2^_(2)_ = 6.22, *p* = 0.04. This distribution of responses corresponded to a weighted average of 25.6° of shift in the perceived location of the flash. In the final trial, when the flash was expected, 169 participants (68.4%) gave the correct response in the central task, and, out of them, 139 (82.2%) reported noticing the flash. Most of the participants who noticed the flash reported perceiving the flash shifted 30° (42.0%), followed by 10° (33.7%) and 50° (6.51%). This distribution of responses also differed from chance, χ^2^_(2)_ = 18.32, *p* = 0.0001, and corresponded to a weighted average of 19.2° of shift in the perceived location.

In the group without the central task, the first presentation of the flash was noticed by 70% of a total of 100 participants. Most of them reported that the flash was shifted 30° (62.9%), with 10° and 50° shifts reported significantly less frequently, 14.3% and 22.9%, respectively; χ^2^_(2)_ = 28.23, *p* < 10^−5^. This distribution of responses corresponded to a weighted average of 31.8° shift. The second presentation of the flash was noticed by 78% of participants, with most reporting a 30° shift of the flash (53.8%), followed by a 10° shift (39.7%) and a 50° shift (6.4%), corresponding to a weighted average of 24.7° of shift.

Two comparisons are of interest here. In both groups, the first presentation of the flash produced a larger flash grab than the second presentation, with the central task: χ^2^_(2)_ = 14.12, *p* = 0.0009; without the central task: χ^2^_(2)_ = 16.18, *p* = 0.0003, confirming the results of the Experiment 1. Critically, in the group with the central task, the first presentation of the flash produced a smaller flash grab than in the group without the central task; fewer participants reported a 30° shift and more participants reported a 10° shift; χ^2^_(2)_ = 20.31, *p* < 10^−4^. The reduction of attention to both the motion and the target for the first, unexpected flash in the group with the central task, produced a decrease in the flash grab effect.

### Discussion

Here we manipulated attention to the target flash and to the background motion with the presence or absence of a secondary task at fixation. The initial flash (of two) was unexpected and as in Mack and Rock's (1998) original inattentional blindness paradigm, more than 60% of participants were unaware of the first flashed target when they were performing the central task. In contrast, without the task at fixation, only 30% of participants failed to notice the unexpected flash. For the second, expected flash, 80% of participants in both conditions noticed the flash. Importantly, the reported location was shifted farther from the actual location when there was no central task; in this case, we assume the background motion and the target both attracted more attention than when there was a central, distracting task. The flash grab effect decreased with this reduction of attention that accompanied the central task. This decrease is consistent with attention to the motion as the causal factor, because decreased attention to the target should have increased the illusion. This work demonstrates more directly that it is the attention to the background motion that increases the effectiveness of the motion-induced position shift.

Our data are taken only from the participants who reported seeing the flash. Of course, if the flash is not seen, there can be no illusion to report. Nevertheless, it is possible that there is an unconscious registration of the flash for the participants who did not report seeing it, and, if position uncertainty were a factor in the strength of the illusion, these unconsciously registered flashes might have a larger illusion size. The location of the unseen flash might be decoded indirectly from functional magnetic resonance imaging (Ge et al., 2020; Kohler, Cavanagh, & Tse, 2017) or electroencephalography signals (Hogendoorn, Verstraten, & Cavanagh, 2015). However, the uncertainty for the flashes that are reported is already at the maximum level that still supports conscious report, and even so the illusion is smaller than in conditions with higher certainty (where there was no central task). We have no reason to imagine that increasing the uncertainty further would reverse this effect and increase the illusion. Indeed, it is the decrease of the illusion with the increasing target uncertainty here that lets us argue that target uncertainty is not the cause of the illusion.

## General discussion

The goal of this study was to determine if target predictability decreases the strength of the flash grab illusion. Experiment 1 showed that the flash grab position shift is as much as 50% larger for targets appearing at unexpected locations than for targets at expected locations. Experiment 2 showed that the flash grab changes with the distribution of attention, with a narrower attention focus, resulting in a smaller illusion. Experiment 3 demonstrated a weaker illusion in the left visual field compared with the right visual field, consistent with the left vs. right asymmetry seen for the flash-grab effect in neglect patients (De Vito et al., 2015). Finally, Experiment 4 showed that, for an unexpected flash, attention to the underlying motion increases the strength of the illusory shift.

We considered three alternatives to explain how attention mediates these reductions for the flash grab effect. The first two are mediated by attention to the target and in both, increased attention to the target will decrease its illusory position shift. The third is based on the effect of attention on the background motion and here, increased attention to the motion will increase the illusory position shift. The below discussion provides more details for these predictions. Note that the effects of attention in opposing directions cannot be distinguished in Experiments 1 and 2 because of the reciprocal relation of attention to the target and attention to the motion: increasing attention to one will decrease it to the other. They are, however, disentangled in Experiment 4, where the data favor attention to the motion as the source of attention's influence on the flash grab illusion.

The first explanation is based on the target uncertainty where decreased target uncertainty will make the target's perceived location less susceptible to the effects of the background motion. Equivalently, decreased target uncertainty will engage attention more strongly to the expected target location, reducing the illusion. This finding is in line with several studies investigating target predictability in motion-induced position shifts. Spatial predictability or cuing of spatial attention attenuates most illusions in this family: the flash lag (Namba & Baldo, 2004; Vreven & Verghese, 2005), the Fröhlich effect (Adamian & Cavanagh, 2017; Müsseler & Aschersleben, 1998), and representational momentum (Hayes & Freyd, 2002). Our results add the flash grab illusion to this list.

In the second explanation, it is the delay of attention in reaching the target that produces the position shift and a shorter delay (when attention is already on the target) therefore decreases this shift. In favor of this alternative, several articles have argued that the delay in shifting attention to a moving object is responsible for flash lag and Fröhlich effects (Adamian & Cavanagh, 2017; Baldo & Namba, 2002; Kirschfeld & Kammer, 1999; Müsseler & Aschersleben, 1998), and it is possible that a similar mechanism is implicated in the flash grab illusion. Attention is necessary to register the location of the object and attention would arrive at the target later if it is initially directed elsewhere or spread more diffusely around it. Critically, during the shift of attention to the moving object, the object continues to move, increasing the mislocalization of its final position when it is acquired. A similar argument has been made for saccades to the flash grab target where participants with slower saccades show a larger perceptual illusion (van Heusden et al., 2018). In this account, anything that speeds the arrival of attention at the target, for example, having a known location, will decrease the illusion. For the flash grab, it is assumed that the stationary flash inherits the motion of the underlying pattern (Cavanagh & Anstis, 2013), and the apparent position of the flash continues to be displaced, consistent with that motion, until the target location is acquired by attention even though the target is no longer present.

Finally, rather than attention to the target, attention to the background motion may be the critical factor affecting the perceive location of the target. In the first two experiments, increased attention to the target location may reciprocally reduce attention to the motion signal, thus reducing the shift the motion would normally produce. Because of this attentional trade-off, these first two experiments could not distinguish between the contribution of attention to the target and attention to the motion. However, results for the flash grab with neglect patients (De Vito et al., 2015) showed that, when attention to both the target and the background motion is decreased in their neglected left visual field, the illusion was reduced. If attention to the target was the critical factor in producing the illusion, then the decrease in attention to the target in the neglected field should have increased the illusion. This finding suggested that, instead, the causal factor was the attention to the motion and with reduce attention to the motion in the neglected field, the illusion decreased. Experiment 4 recreated the neglect effect in healthy participants by adding a central task and making the target appearance completely unexpected. These conditions insured a reduced level of attention to both the target and the motion. As with the neglect patients, the illusion was reduced by this withdrawal of attention to both target and motion. According to the first two explanations above, decrease of attention to the target should increase the flash grab effect but instead, it decreased. This result indicated that the attention to the motion was driving the illusory shift, not attention to the target. This explanation does not provide a mechanism by which motion produces the shift, it only specifies that whatever that mechanism, its effect will decrease when the attention to the motion is reduced.

Our results suggest that attention to the background motion dominates attention's contribution to the illusion. This finding does not rule out a smaller (and opposite) contribution from attention to the target, but to test this experimentally, we would need a task in which attention to the background motion would be held constant while attention to the target was varied. This experiment would be a challenge to develop. Moreover, it is possible that the intensity of attention's focus on the target is generated principally by the exogenous attention attracted to the flash so that the manipulation of endogenous or sustained attention to the flash location in our experiments acted mainly to deprive motion of attention and did little to increase the attention to the target. Earlier studies had already shown effects of attention to the motion for the flash grab illusion (Cavanagh & Anstis, 2013; Tse et al., 2011) and our original goal here was to extend these results and evaluate the effect of attention to the target. Ironically, in the end, the results pointed back to the attention to the motion as the primary factor. Specifically, when the potential effects of attention to the target and attention to the motion are tested together (Experiment 4), the attention to the motion dominates. Note however, that when attention was distracted from both the target and the motion, the illusory effect decreased by only approximately 15%—it did not disappear. Clearly, some attention to the motion was maintained, even with the central task and that residual amount of attention would be reasonable, given that motion is a strong attractor of attention, even when it is irrelevant to the participants’ task.

Does this primacy of the attention to the motion help us understand the source of this motion-induced position shift? First, the results argue against either the uncertainty of the target location or the delay of attention in reaching the target as the principal factor in producing the effect. Motion itself may cause the position shift owing to position averaging that shortens the motion path (Krekelberg & Lappe, 2000; Takao, Sarodo, Anstis, Watanabe, & Cavanagh, 2022). In this case, this path shortening operates only on attended motion trajectories. Alternatively, the position shift may be caused by attentional repulsion that expands space around the focus of attention and shifts nearby items away (Shams-Ahmar, Kohler, & Cavanagh, 2023; Suzuki & Cavanagh, 1997). In this case, the role of attention is self-evident.

## Supplementary Material

Supplement 1

## Supplementary material

Supplementary Movie S1. Example of flash grab with *1/f* texture and the flash in an unexpected location. The textured annulus rotates back and forth and the green disc flashes at one of the reversals on either the left, right, top, or bottom. It then flashes a second time in the same location. Please maintain fixation on the central dot. You may notice an offset between the flash and its actual location (always 0°, 90°, 180°, or 270° from vertical) in the direction of the following motion, always counterclockwise here. You may also notice that the second flash has less of an offset. If so, your observations match those of our participants. https://cavlab.net/Demos/FGMovie1.
